# Comparative Genomics Analysis of Habitat Adaptation by *Lactobacillus kefiranofaciens*

**DOI:** 10.3390/foods12081606

**Published:** 2023-04-10

**Authors:** Rui Luo, Chen Liu, Yu Li, Qing Liu, Xin Su, Qingting Peng, Xueyan Lei, Weicheng Li, Bilige Menghe, Qiuhua Bao, Wenjun Liu

**Affiliations:** 1 Key Laboratory of Dairy Biotechnology and Engineering (IMAU), Ministry of Education, Inner Mongolia Agricultural University, Hohhot 010018, China; 2 Key Laboratory of Dairy Products Processing, Ministry of Agriculture and Rural Affairs, Inner Mongolia Agricultural University, Hohhot, 010018, China; 3 Inner Mongolia Key Laboratory of Dairy Biotechnology and Engineering, Inner Mongolia Agricultural University, Huhhot 010018, China; 4 Collaborative Innovative Center of Ministry of Education for Lactic Acid Bacteria and Fermented Dairy Products, Inner Mongolia Agricultural University, Hohhot, 010018, China

**Keywords:** *Lactobacillus kefiranofaciens*, comparative genomics, habitat adaptation

## Abstract

*Lactobacillus kefiranofaciens* is often found in fermented dairy products. Many strains of this species have probiotic properties, contributing to the regulation of immune metabolism and intestinal flora. This species was added to the list of lactic acid bacteria that can be added to food in China, in 2020. However, research on the genomics of this species is scarce. In this study we undertook whole genome sequencing analysis of 82 strains of *L. kefiranofaciens* from different habitats, of which 9 strains were downloaded from the NCBI RefSeq (National Center for Biotechnology Information RefSeq). The mean genome size of the 82 strains was 2.05 ± 0.25 Mbp, and the mean DNA G + C content was 37.47 ± 0.42%. The phylogenetic evolutionary tree for the core genes showed that all strains belonged to five clades with clear aggregation in relation to the isolation habitat; this indicated that the genetic evolution of *L. kefiranofaciens* was correlated to the isolation habitat. Analysis of the annotation results identified differences in the functional genes, carbohydrate active enzymes (CAZy) and bacteriocins amongst different isolated strains, which were related to the environment. Isolates from kefir grains had more enzymes for cellulose metabolism and a better ability to use vegetative substrates for fermentation, which could be used in feed production. Isolates from kefir grains also had fewer kinds of bacteriocin than isolates from sour milk and koumiss; helveticin J and lanthipeptide class I were not found in the isolates from kefir grains. The genomic characteristics and evolutionary process of *L. kefiranofaciens* were analyzed by comparative genomics and this paper explored the differences in the functional genes amongst the strains, aiming to provide a theoretical basis for the research and development of *L. kefiranofaciens*.

## 1. Introduction

Kefir is a viscous, slightly sour dairy product that is made from fermented kefir grains [1] and has many health benefits [2,3]. Kefir grains were first described by the tribes in the northern Caucasus mountain region of Russia [4]. They contain a diverse range of lactic acid bacteria, yeast and sometimes acetic acid bacteria in a polysaccharide matrix of semi-hard granules. *Lactobacillus kefiranofaciens* is a major microbial constituent of kefir grains and forms highly viscous colonies [5,6].

In 1986, Toba [7] used KPL agar medium to isolate *Limosilactobacillus fermentum* strains from kefir. Some strains were markedly different from previously described species and had capsular membranes. In 1988, Fujisawa [8] described the characteristics of these strains and named them *L. kefiranofaciens.* In 2004, Vancanneyt [9] divided the strains into two subspecies according to their morphological and phenotypic characteristics, namely *L*. *kefiranofaciens* subsp. *kefiranofaciens* and *L*. *kefiranofaciens* subsp. *kefirgranum*. In 2020, the genus *Lactobacillus* was reclassified and redescribed; *L. kefiranofaciens* is still classified into the revised genus *Lactobacillus* [10]. In December 2020, China’s Health Commission designated *L. kefiranofaciens* subsp. *kefiranofaciens* as a new raw food material that could be added to other foods. Many studies have shown that *L. kefiranofaciens* has probiotic properties, for e.g., the regulation of immune metabolism [11], the regulation of intestinal microorganisms [12], anti-allergy properties [13] and antibacterial properties [14].

In recent years, comparative genomics has been widely used in research on lactic acid bacteria. A large number of lactic acid bacteria have had their whole gene sequenced. Through comparative genomics analysis the differences in genome structure, quantity and function between species or between different individuals of the same species can be identified [15]. This can reveal the relationship between genetic evolution and physiological characteristics of strains [16] and how the environment impacts growth [17]. Anxiaona [18] used comparative genomics to evaluate habitat adaptability in 34 strains of *Limosilactobacillus reuteri* from different sources and found that the genes of *L. reuteri* from different sources are diverse and closely related to the habitat in which the strain has been living, and that the number and types of carbohydrate and amino acid-related genes varied greatly amongst strains isolated from different habitats. Broadbent [19] evaluated *Lacticaseibacillus casei* isolated from different habitats using comparative genomics and found that there were particular genes in the genome of *L*. *casei* from different isolation sources that were related to their living environment. In contrast, Verce Marko [20] found that *L*. *fermentum* isolated from different sources exhibited no clear strain clustering, indicating that the lifestyle of *L*. *fermentum* is less specialized. In 2011, the genome of the first strain of *L. kefiranofaciens* ZW3 was sequenced [21]. In 2017, Zhuqing Xing [22] conducted comparative genomic analysis of ZW3 and five other closely related *Lactobacillus* strains, and found a series of genes related to the dairy environment and the intestinal environment.

Comparative genomics research on homologous bacteria provides information about the changes, deletions or acquisitions of genes that could promote the evolution of strains in different niches and enhance environmental adaptability [23]. In order to further understand the habitat adaptability of *L. kefiranofaciens*, 73 new isolates and 9 strains downloaded from the NCBI were compared using comparative genomics analysis to evaluate their genetic diversity and habitat adaptability, and to reveal the mechanisms of habitat adaptability in different living environments at the genetic level. The results of this study provide a theoretical basis for selecting *L. kefiranofaciens* with prebiotic functions.

## 2. Material and Methods

### 2.1. Strains

A total of 82 strains of *L. kefiranofaciens* were evaluated in this study. Nine of these were from the NCBI RefSeq (National Center for Biotechnology Information RefSeq. https://www.ncbi.nlm.nih.gov/, accessed on 18 March 2022), NCBI published the full gene sequence of these nine strains of *L. kefiranofaciens*, Table 1). The remaining 73 strains were preserved by freeze drying and held in the Lactic Acid Bacteria Collection Center (LABCC), Inner Mongolia Agricultural University.

### 2.2. DNA Extraction

Freeze-dried material from the 73 strains of *L. kefiranofaciens* were inoculated in a Man–Rogosa–Sharpe (MRS) medium and cultured for three generations. The DNA was extracted using the TIANGEN bacterial genomic DNA kit (China TIANGEN Corporation), according to the manufacturer’s instructions.

### 2.3. DNA Quality Control and Genome Resequencing

The extracted DNA was sent to Novo Biogenics for sequencing using the Illumina NovaSeq 6000 high-throughput sequencing platform. Moreover, 150 bp was selected to construct paired-end (PE) sequencing libraries and the average coverage of high-quality data was about 500 X.

### 2.4. Genome Splicing Assembly

Clean data was obtained by filtering raw data. SOAP (denovo version 2.0, HKU-BGI Bioinformatics Algorithms and Core Technology Research Laboratory & Department of Computer Science, University of Hong Kong) was used for splicing and assembly of high-quality reads and appropriate kmer values were selected [24]. The filtered data were spliced and assembled with single base corrections.

### 2.5. Comparative Genomics Analysis

#### 2.5.1. Average Nucleotide Identity (ANI) Analyses

The ANI values of all 82 strains were calculated using the self-made Perl script, according to the method reported by Goris [25]. The software TBtools (State Key Laboratory for Conservation and Utilization of Agro-Biological Resources in Subtropical Region, South China Agricultural University, College of Horticulture, South China Agricultural University, Guangzhou, China) [26] was used to draw the cluster heat map.

#### 2.5.2. Construction of Pan-Core Gene Sets

Prokka software (University of Melbourne, Victoria, Australia) [27] was used to predict the genes present in the 82 *L. kefiranofaciens* strains and Roary software (Pathogen Genomics, The Wellcome Trust Sanger Institute, Wellcome Trust Genome Campus, Hinxton, Cambridge, UK) [28] was used to identify and count the core genes and pan-gene sets. The gene families were divided according to the standard of amino acid consistency greater than 90%, and the core gene sets and pan-gene sets were constructed. The pan-genome refers to all the genes contained in these strains, the core gene refers to the genes shared by these strains, and unique genes were those that appeared in the genome of only a single strain.

#### 2.5.3. Construction of Phylogenetic Trees

The phylogenetic tree was constructed based on the core gene set using treebest (http://www.mybiosoftware.com/treebest. accessed on 20 March 2022) and the neighbor-joining (NJ) method. Visualization was performed using the iTol online software (https://itol.embl.de/. accessed on 20 March 2022).

#### 2.5.4. Functional Gene Annotation

Genome annotation of the 82 strains was achieved using the RAST website (Rapid Annotation using Subsystem Technology, http://rast.nmpdr.org/rast.cgi, accessed on 24 March 2022) and compared with the Clusters of Orthologous Groups of Proteins (COGs) database.

#### 2.5.5. Carbohydrate Active Enzyme Analysis

We used dbCAN2 (http://bcb.unl.edu/dbCAN2/. accessed on 27 March 2022) to identify the active genes for the enzymes of carbohydrates in the 82 strains of *L. kefiranofaciens* [29]. The strain sequences were analyzed by combining them with the CAZy (carbohydrate-active enzymes).

#### 2.5.6. Mapping and Data Analysis

SPSS software (IBM, Armonk, New York, NY, USA) was used to calculate the significance of differences in x amongst the strains and the GraphPad Prism 9.0 software (GmphPd Software inc, San Diego, CA, USA) was used to draw graphs.

## 3. Results

### 3.1. General Genomic Characteristics of L. kefiranofaciens

The average genome size of *L. kefiranofaciens* was 2.05 ± 0.25 Mbp; the maximum value was 2.30 Mbp (strain RU15-4) and the minimum value was 1.86 Mbp (ELS7-3). The range of variation in G + C content was relatively stable; the maximum value was 37.78% (strain F3301) and the minimum value was 37.05% (strain RU15-4), with the average value being 37.47 ± 0.42% (Table S1 and Table 1).

### 3.2. Average Nucleotide Identity (ANI) Analysis

**Figure 1:**
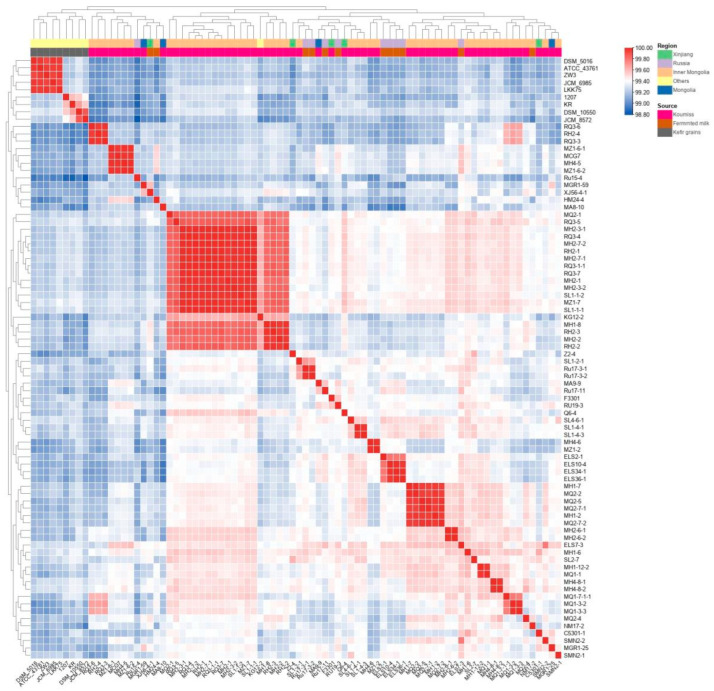
ANI calorific diagram of 82 *L. kefiranofaciens* strains.

The ANI values of the 82 *L. kefiranofaciens* strains were calculated and a clustering heat map constructed (Figure 1). This showed that the ANI values of all strains were above 98.7%, which demonstrates that all strains were the same species, and that intraspecific homology was high. The ANI values of six strains (MH 1-7, MQ 2-2, MQ 2-5, MQ 2-7-1, MH 1-2, MQ 2-7-2) identified as subspecies by the NCBI were high and clustered together (Figure 1). Most strains isolated from Inner Mongolia (upper orange band) showed a certain clustering trend and strains from other regions were distributed amongst them. The inner source band showed that strains from kefir grains were clustered into one group (gray band) and that strains from sour milk and koumiss were staggered and clustered. According to the ANI value heat map, it can be inferred that the clustering of *L. kefiranofaciens* has some relationship with the source and location of the isolation.

### 3.3. Phylogenetic Tree Construction Based on Core Genes

**Figure 2:**
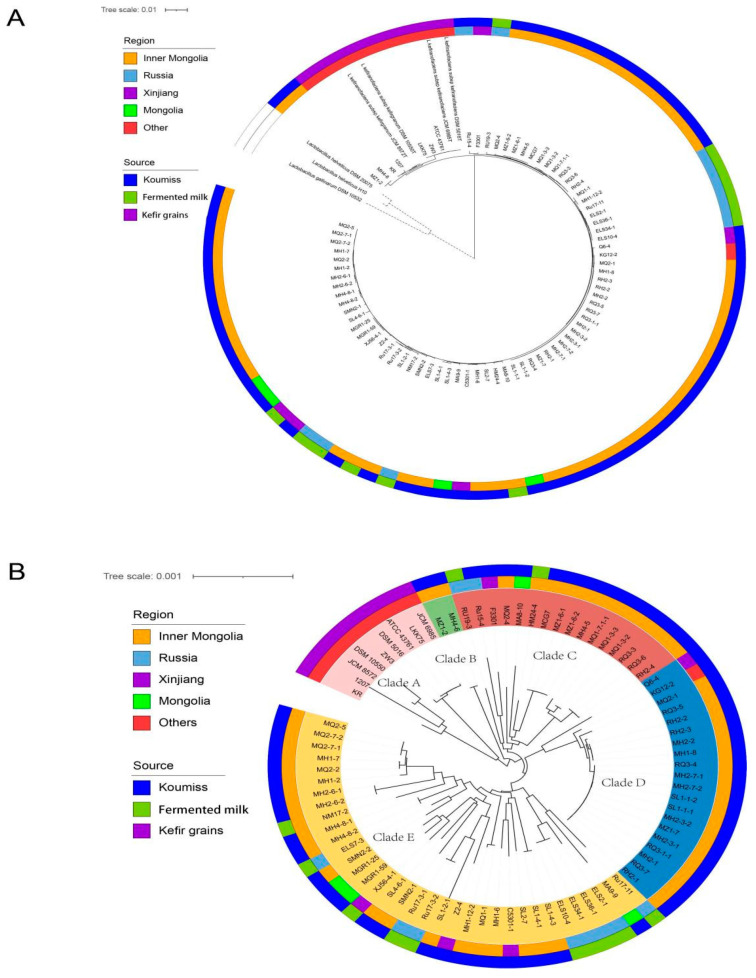
Phylogenetic trees constructed based on 1045 core genes in 82 *L. kefiranofaciens* strains. Constructing phylogenetic tree based on 1045 core genes of 82 strains of *Lactobacillus kefiranofaciens*, The *Lactobacillus helveticus* DSM 20075^T^, the *Lactobacillus helveticus* H10 and the *Lactobacillus gallinarum* DSM 10532^T^ were used as outgroups (**A**). (**B**) depicts the isolation information of 82 strains, with the inner layer marking the location of strain isolation and the outer layer marking the source of strain isolation.

The phylogenetic tree constructed based on 16S rRNA showed that the genetic distance between most strains could not be determined and that type strains for two subspecies clustered in the same branch and could not be distinguished (*L. kefiranofaciens* subsp. *Kefiranofaciens* type strain: ATCC 43761 = DSM 5016 = JCM 6985, *L. kefiranofaciens* subsp. *Kefirgranum* type strain: DSM 10550 = JCM 8572) [30]. In order to determine the evolutionary relationships amongst the strains, a phylogenetic tree was constructed based on 1045 core genes in the 82 strains of *L. kefiranofaciens*; *Lactobacillus helveticus* DSM 20075^T^, *L. helveticus* H10 and *Lactobacillus gallinarum* DSM 10532^T^ were used as outgroups (Figure 2A). The sources from which the 82 strains were isolated are shown in Figure 2B. The 82 strains of *L. kefiranofaciens* could be divided into five evolutionary clades. The nine strains isolated from kefir grains (all from the NCBI) were clustered in clade A, which was close to the common ancestor and had a close genetic distance. Strains isolated from Inner Mongolia Xilin Gol koumiss (MZ1-2, MH4-6) clustered in clade B. Branch C contained 16 strains including isolates from Russian koumiss and sour milk, Xinjiang koumiss, Mongolian koumiss and Inner Mongolia Xilin Gol koumiss. There were 20 strains in clade D (including strains from Xinjiang koumiss, Uzbekistan koumiss and Inner Mongolian koumiss). The geographical locations and isolation sources of clade E strains were complex, most were isolated from Inner Mongolia and Russia and some strains showed a clustering trend.

These results indicated that *L. kefiranofaciens* strains showed a trend to cluster in relation to the source from which they were isolated, and that the evolution of *L. kefiranofaciens* was correlated with the source habitat. In addition, we found that we could not distinguish the subspecies status of the strains using the phylogenetic tree constructed from the core genes.

### 3.4. Gene Prediction and Annotation

The functional genes of the 82 *L. kefiranofaciens* strains were predicted and annotated by RAST. A total of 25 functional categories were annotated. The genes related to carbohydrate metabolism accounted for the highest proportion in *L. kefiranofaciens* (15.42%), followed by genes related to protein metabolism (13.99%), amino acids and their derivatives (11.30%) and nucleosides and nucleotides (9.67%) (Figure S1). These results indicate that these functional genes were indispensable for strain growth. The relative proportions of the functional genes of each type varied amongst the strains. In other studies, the *L. helveticus* strain had the largest proportion of functional genes related to protein metabolism [31] and this strain also had a strong capacity for carbohydrate metabolism.

The COG database was used to carry out the same functional annotation as shown in Figure S2. Each strain contained 1843 coding genes, on average. Amongst the 82 *L. kefiranofaciens* strains, genes involved in life activities and information storage accounted for the highest proportion, for e.g., translation, ribosome structure and biogenesis, transcription, replication, recombination and repair. This was followed by genes involved in metabolism, including carbohydrate transport and metabolism, amino acid transport and metabolism, and nucleotide transport and metabolism; genes with unknown function also represented a high proportion of the total genes.

### 3.5. Core Genome and Pan-Genome Analysis

The core genome and pan-genome of the 82 *L. kefiranofaciens* strains were determined by comparative genomics. The core genome contained 1045 gene families and the curve of the core genes tended to be stable, indicating that the number of core genes remained basically unchanged with the increasing number of strains, and that the gene set of these strains were open genes. The pan-genome contained 7189 gene families and the curve was not stable, but showed an upward trend. With increasing numbers of strains the number of pan-genes continued to increase.

**Figure 3:**
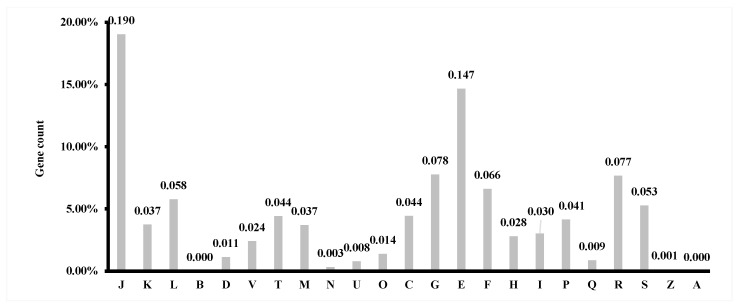
Annotation of a core genome based on the COG database. A: RNA processing and modification, B: Chromatin structure and dynamics, C: energy production and conversion, D: cell cycle control, cell division, chromosome partitioning, E: amino acid transport and metabolism, F: nucleotide transport and metabolism, G: carbohydrate transport and metabolism, H: coenzyme transport and metabolism, I: lipid transport and metabolism, J: translation, ribosomal structure and biogenesis, K: transcription, L: replication, recombination and repair, M: cell wall/membrane/envelope biogenesis, N: cell motility, O: post-translational modification, protein turnover, P: inorganic ion transport and metabolism, Q: secondary metabolites biosynthesis, transport and catabolism, R: General function prediction only, S: function unknown, T: signal transduction mechanisms, U: intracellular trafficking, secretion and vesicular, V: defense mechanisms, Z: Cytoskeleton.

The COG annotation of the core genome showed that 28.54% of the genes contributed to vital activities of the strains, predominantly translation, ribosome structure and biogenesis (19.03%), carbohydrate metabolism (14.67%), signal transduction, energy generation and transformation, and inorganic salt ion transport (12.88%) (Figure 3). The results largely confirmed the previous overall annotation results and indicated that the function of the core genes was essential for the life of these strains.

The presence or absence of accessory genes in all strains can be seen in Figure S3. Blue represents the presence of a gene, while white represents the absence of a gene. The presence of accessory genes was related to strain habitat, and the distribution of accessory genes was similar in strains from a similar origin. Gene presence and deletion also showed that the gene distribution in strains from kefir grains was similar to that of other sources.

### 3.6. Analysis of Differences Amongst Isolates

**Figure 4:**
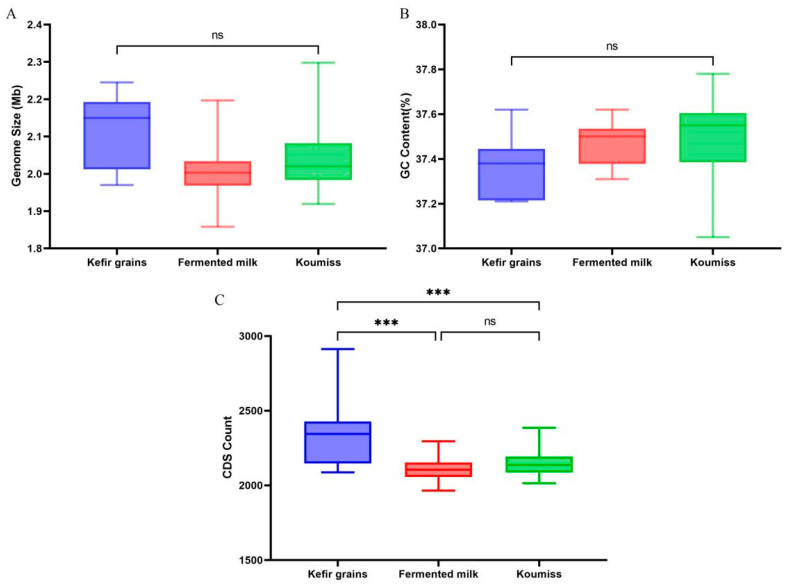
Genomic characteristics of *L. kefiranofaciens* strains isolated from different sources. ***: Samples have a very large significant difference. ns: Samples have no significant difference. (**A**): Genome size of different isolates. (**B**): GC content of different isolates. (**C**): CDS count of different isolates.

According to the previous analyses, strains isolated from the same source clustered together. We then compared the strains isolated from different sources (koumiss, sour milk, kefir grains); there was a significant difference in the number of CDS (coding sequence) (*p* < 0.001) and strains from kefir grains had more CDS than strains from koumiss or sour milk (Figure 4).

**Figure 5:**
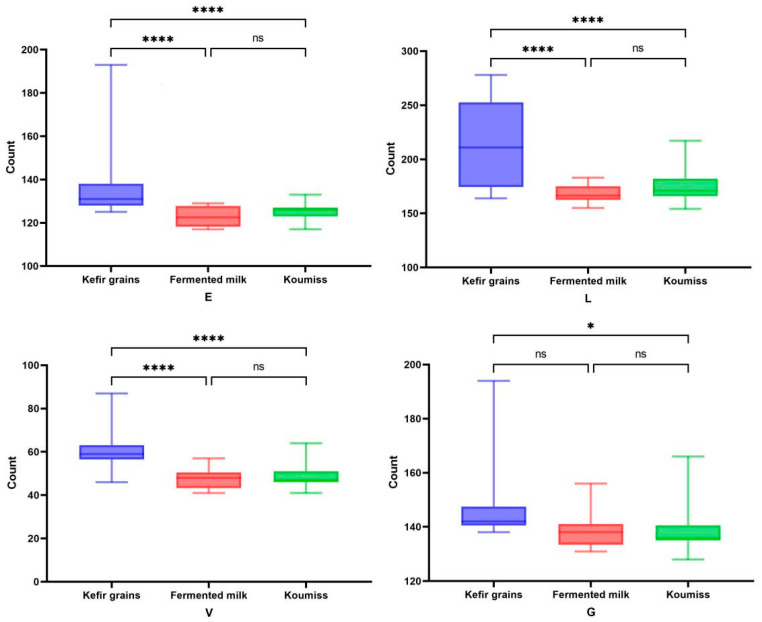
Differences in COG function annotation of different *L. kefiranofaciens* strains. *: Samples have a little significant difference. ****: Samples have a large significant difference. ns: Samples have no significant difference. E: Amino acid transport and metabolism. G: carbohydrate transport and metabolism. L: replication, recombination and repair. V: Defense mechanism.

The COG annotation analysis of the functional genes in strains isolated from different sources showed that the differences between the strains from kefir grains, koumiss and sour milk were mainly in those related to amino acid transport and metabolism (E), replication and recombination repair (L) and defense mechanisms (V). Strains from kefir grains had significantly more of these three types of genes than strains from the other two sources; this indicates that strains from kefir grains were superior to the koumiss and sour milk strains in terms of amino acid metabolism and defense mechanisms (Figure 5). There was a significant difference in the genes related to carbohydrate metabolism in strains from koumiss and kefir grains (*p* < 0.05).

**Figure 6:**
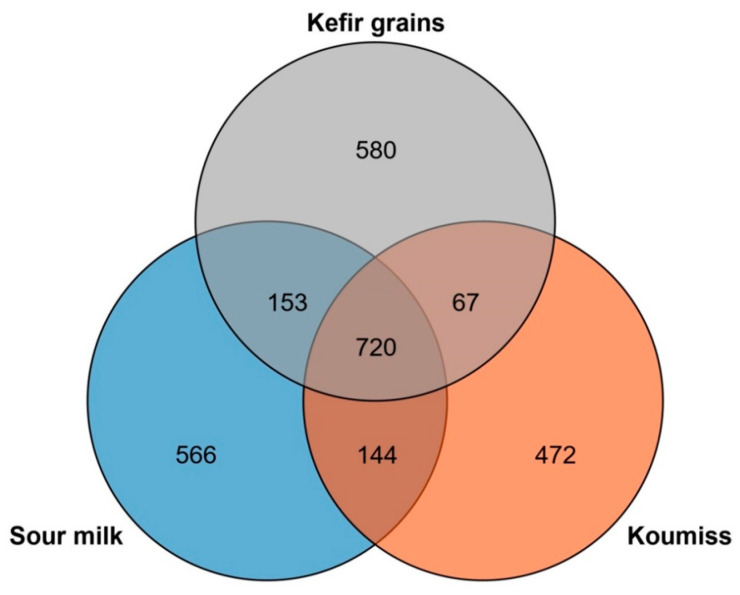
Venn diagrams of core gene sets of *L. kefiranofaciens* strains isolated from different sources.

The trend in the COG annotation results was basically the same as that for core gene annotation; there was still a 30% difference in the core genes between the strains isolated from different sources (Figure 6). The amino acid transport and metabolism (E) and translation, ribosome structure and biogenesis (J) genes were lower in strains isolated from kefir than from those isolated from koumiss and sour milk. Strains from koumiss were lower in genes relating to energy production and conversion (C), carbohydrate transport and metabolism (G) and replication, recombination and repair (L) than the other strains, while strains from sour milk were lower in genes relating to nucleotide transport and metabolism (F). These results indicate that the environment that strains were living in affected the number of genes involved in major life activities. At the same time, there were fewer core genes in strains from koumiss, indicating that the genetic diversity of these strains was higher than in strains from other sources. The proportion of unique genes in strains from kefir grains and sour milk strains was high, indicating that the individual differences between strains were large, which was related to their geographical distance apart. However, the geographical areas of the koumiss strains were concentrated, indicating that the individual differences were small.

After removing the putative protein (unknown function) from the unique genes of the strains, there were 351 functional genes actually encoded by the strains isolated from kefir grains, including amino acid biosynthesis, monosaccharide metabolism and the CRISPR-Cas immune defense mechanism. The strains isolated from koumiss actually encoded 133 functional genes, which were mainly involved in protein metabolism and synthesis, the ABC transport system and various secreted proteins. There were 145 unique functional genes in the strains from sour milk, mainly encoding lactose and galactose metabolism, specific PTS (phosphotransferase system) transport of various monosaccharides (sorbitose, fructose, tagatose, mannose, ascorbic acid), amino acid metabolism and transposons.

The lack of unique genes in the strains from koumiss may be related to the close distance between the strains. The 35 strains in this group had no unique genes, all of which were isolated from strains from Xilin Gol, Inner Mongolia, some of which had unique genes for sugar metabolism (such as maltose and cellobiose, etc.). Koumiss is rich in protein and high in free amino acids, especially arginine and cysteine, so strains isolated from koumiss had many genes related to protein metabolism. There were more unique genes for lactose and galactose utilization in the sour milk strains, and more lactose metabolism genes were found in the strains from sour milk with higher lactose content. There were differences in the unique genes amongst the three sources. However, because the strains were all from dairy products, there were similar genes for protein and lactose metabolism.

### 3.7. CAZy Analysis

**Figure 7:**
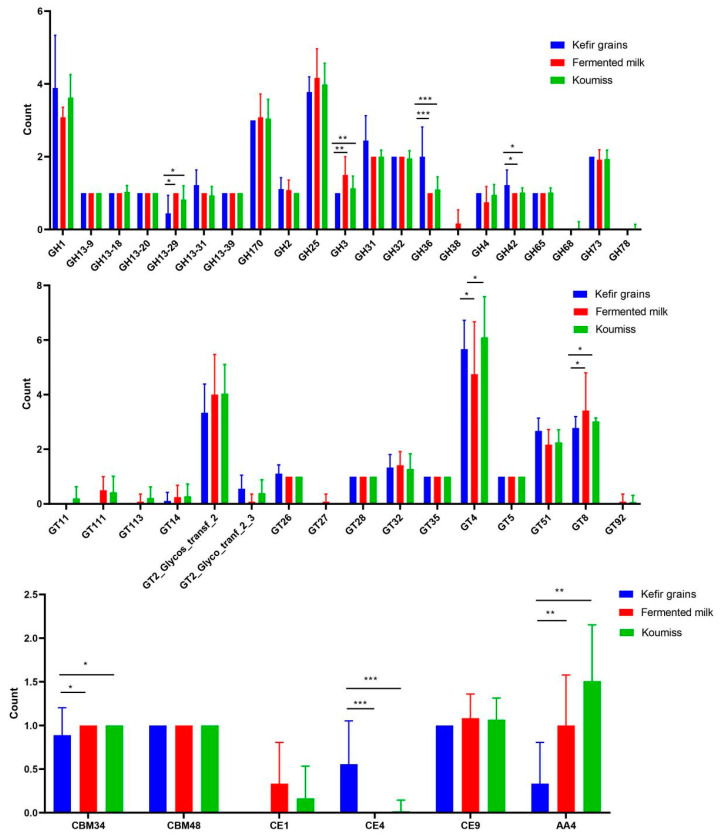
Differences in CAZy in different *L. kefiranofaciens* strains isolated from different sources. *: Samples have a little significant difference. **: Samples have a large significant difference. ***: Samples have a very large significant difference.

The 82 *L. kefiranofaciens* strains were annotated; there were 43 functional subclasses, mainly glycosyltransferase (GT), glycoside hydrolase (GH) and carbohydrate esterase (CE). The strains from different habitats showed significant differences in enzymes, for e.g., GH13_29, GH42, GH3, GH31, GH36, GT8 and CE4 (Figure 7). The strains from kefir grains had significantly higher levels of GH31, GH36 and GH42 enzymes involved in cellulose metabolism compared with strains from koumiss and sour milk. At the same time, the strains from kefir grains also had enzymes from the CE4 family, including acetyl xylan esterases and chitin deacetylase, which hydrolyze hemicellulose and degrade wood fibers [32]. Therefore, we speculate that the strains from kefir grains can ferment plant substrates to a certain extent and have better cellulose metabolism ability. However, enzymes from the AA4 family were more common in the strains from koumiss and sour milk. This family encodes vanillin alcohol oxidase, which can catalyze the conversion of various phenolic compounds of aromatic ring paraposition side chains, catabolizes aromatic phenols and converts lignin-derived aromatic monomers into valuable compounds [33].

### 3.8. Bacteriocin Analysis

In this study, 273 operons of bacteriocins were predicted from 82 genomes, including 26 class I bacteriocins and 249 class III bacteriocins. In addition to the two strains without the bacteriocin operon, enterolysin A and helveticin J of class III bacteriocins were predicted in all the other strains, indicating that these two bacteriocins were common in this strain. According to previous studies, horizontal gene transfer can occur in bacteriocin helveticin J, which was found in six strains related to *Lactobacillus acidophilus* [34], while helveticin J was only found in some strains from koumiss and sour milk. According to previous studies, class III bacteriocins are heat unstable and mainly appear in *Lactobacillus* strains. Helveticin J has been studied more, and its mechanism of action is bactericidal, but its antibacterial spectrum is narrow [35]. The antibacterial spectrum of enterolysin A is relatively broad [36].

**Figure 8:**
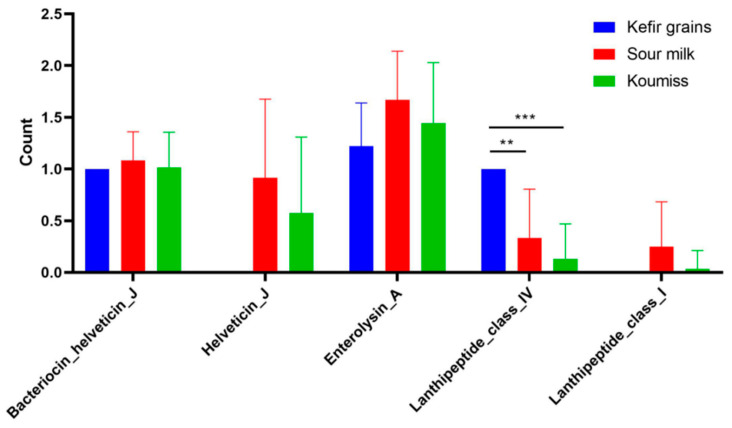
Difference analysis of bacteriocins produced by *L. kefiranofaciens* strains isolated from different sources. **: Samples have a large significant difference. ***: Samples have a very large significant difference.

There were differences in bacteriocin distribution in the strains isolated from different sources. With the exception of enterolysin A and bacteriocin helveticin J, the other three bacteriocins were all different. Helveticin J and lanthipeptide class I did not appeared in strains from kefir grains but did appear in some strains from koumiss and sour milk. Lanthipeptide class IV appeared in all strains from kefir grains, but less in strains from sour milk and koumiss (Figure 8).

## 4. Discussion

In this study, the genomic characteristics, pan-gene sets and core gene sets of 82 *L. kefiranofaciens* strains from koumiss, sour milk and kefir grains were compared. A total of 1045 core genes and 7189 pan-genes were found. The pan-genome was open, while the core genome was stable. The genotypes of *L. kefiranofaciens* were generally open, indicating that *L. kefiranofaciens* could exchange genes from the external environment to adapt to that environment. The phylogenetic tree was constructed based on the core genes and ANI values.

The genomic characteristics of the *L. kefiranofaciens* strains from different sources showed differences in the overall functional genes, core genes and unique genes. Among the coding of functional genes, the number of genes related to amino acid transport metabolism and defense mechanisms in the strains from kefir grains were significantly higher than in strains from the other two sources, and there were also differences among the unique genes of each isolate. Differences in protein metabolism, monosaccharide utilization and lactose and galactose metabolism between the strains from koumiss and sour milk may be due to differences in the nutrient composition of the different milk sources [37]. These results also show that the functional genes of *L. kefiranofaciens* in particular living environments function differently in their hosts.

Carbohydrates are the main constituents of the cells, the main energy source and play a regulatory role in life activities [38]. Annotation of the CAZy showed that there were also differences in the CAZy between the strains from different habitats, and the differences between the strains from kefir grains, sour milk and koumiss were very obvious. The strains from kefir mainly had enzymes related to the utilization of cellulose, starch and lactose. It is speculated that this strain has a strong capacity to ferment plant material, which may be related to its complex living environment. Compared with strains from kefir grains, the strains from the other two fermented milk sources had fewer unique genes. The strains from kefir grains were close to the root of the phylogenetic tree, and so the other strains may have originated from kefir grains, which may have lost some genes in the evolution process and evolved to be more adapted to the environment of a single milk source. The presence of the bacteriocin operon predicted suggests that the strain has antibacterial ability and many phenotypic and animal tests have shown that this is associated with an inhibitory ability against pathogenic bacteria [39]. The distribution of bacteriocins in different strains was also different. Lanthipeptide class I did not exist in the strains from kefir grains, but lanthipeptide class IV can be found and was abundant.

In conclusion, the strains of *L. kefiranofaciens* from different sources have a wide range of gene diversity, which is closely related to their living environment, which lays a foundation for exploring the genetic basis and molecular evolution rules for *L. kefiranofaciens* adaptation to different habitats. As more *L. kefiranofaciens* strains are sequenced, more *L. kefiranofaciens* genomes from different environments can be compared, which will provide a strong indication of the factors affecting their genetic diversity and habitat adaptation. Our results provide a theoretical basis for the development of *L. kefiranofaciens*.

## Figures and Tables

**Table 1:** Genome information for nine *L. kefiranofaciens* strains as published by NCBI.

Strains	Isolation Source	Region	Genome Size (Mbp)	GC Content (%)	CDS	tRNA	Accession No.
1207	Kefir grains	Unknown	2.07	37.62	2156	63	GCA_014656585.1
ATCC 43761	Kefir grains	Denmark	2.18	37.21	2344	59	GCA_900103655.1
DSM 10550	Kefir grains	Japan	1.99	37.47	2087	56	GCA_001434195.1
DSM 5016	Kefir grains	Denmark	2.15	37.22	2310	46	GCA_001435275.1
JCM 6985	Kefir grains	Denmark	2.15	37.21	2435	50	GCA_000615685.1
JCM 8572	Kefir grains	Japan	1.97	37.42	2913	53	GCA_001311335.1
KR	Kefir grains	Germany	2.03	37.4	2138	59	GCA_002276565.1
LKK75	Kefir grains	Unknown	2.21	37.38	2385	63	GCA_009184665.1
ZW3	Kefir grains	Tibet	2.25	37.36	2420	61	GCA_000214785.1

GC Content: The ratio of guanine and cytosine.

## Data Availability

Data is contained within the article or supplementary material.

## Supplementary Materials

The following supporting information can be downloaded at: https://www.mdpi.com/article/10.3390/foods12081606/s1, Table S1: Genomic characteristics of 73 strains of *L. kefiranofaciens*; Figure S1. RAST annotation results for 82 strains of *L. kefiranofaciens*; Figure S2. COG annotation results of 82 strains of *L. kefiranofaciens*; Figure S3. Presence and absence of accessory genes in 82 strains of *L. kefiranofaciens.*
