# Supplementary material for: Comparative Genomics Analysis of Habitat Adaptation by Lactobacillus kefiranofaciens
Source: Foods. 2023 Apr 10;12(8):1606. doi: 10.3390/foods12081606 (PMC10137885; doi:10.3390/foods12081606)
Supplement: Supplementary file 1 [file foods-12-01606-s001.zip › foods-2281794-supplementary.pdf]

Supplementary

Table S1. Genomic characteristics of 73 strains of *L. kefiranofaciens*.

Strains	Genome Size (Mbp)	GC Content (%)	CDS	rRNA	tRNA
C5301-1	2.05	37.49	2151	3	52
ELS10-4	2.04	37.48	2165	3	53
ELS2-1	2.00	37.43	2097	3	52
ELS34-1	2.01	37.5	2117	4	53
ELS36-1	2.01	37.5	2113	3	52
ELS7-3	1.86	37.61	1964	0	50
F3301	2.01	37.78	2154	3	58
HM24-4	2.01	37.36	2113	3	52
KG12-2	2.25	37.15	2359	3	43
MA8-10	2.26	37.21	2359	3	53
MA9-9	1.97	37.67	2107	4	50
MCG7	2.10	37.39	2206	3	57
MGR1-25	2.03	37.59	2136	3	52
MGR1-59	2.28	37.13	2380	3	53
MH1-12-2	1.99	37.55	2089	3	55
MH1-2	1.99	37.51	2092	3	52
MH1-6	1.92	37.67	2026	3	52
MH1-7	2.01	37.52	2098	3	56
MH1-8	2.20	37.31	2307	3	56
MH2-1	2.02	37.6	2318	3	52
MH2-2	2.21	37.3	2318	3	52
MH2-3-1	2.06	37.6	2188	3	56
MH2-3-2	2.05	37.61	2187	3	52
MH2-6-1	1.94	37.54	2015	3	52
MH2-6-2	1.94	37.55	2014	3	52
MH2-7-1	2.06	37.61	2187	3	56

Strains	Genome Size (Mbp)	GC Content (%)	CDS	rRNA	tRNA
MH2-7-2	2.06	37.6	2187	3	53
MH4-5	2.09	37.37	2195	3	53
MH4-6	2.04	37.47	2160	3	53
MH4-8-1	1.93	37.6	2019	3	56
MH4-8-2	1.92	37.6	2016	3	53
MQ1-1	1.97	37.5	2068	3	52
MQ1-3-2	1.97	37.59	2106	3	53
MQ1-3-3	1.96	37.59	2099	3	50
MQ1-7-1-1	1.97	37.56	2057	3	52
MQ2-1	1.99	37.61	2091	3	52
MQ2-2	2.01	37.52	2100	3	56
MQ2-4	2.16	37.22	2230	3	50
MQ2-5	2.00	37.5	2081	3	53
MQ2-7-1	1.99	37.5	2072	4	52
MQ2-7-2	2.01	37.51	2082	3	56
MZ1-2	2.05	37.46	2169	3	53
MZ1-6-1	2.08	37.37	2175	3	53
MZ1-6-2	2.09	37.38	2188	3	53
MZ1-7	1.98	37.63	2093	3	56
NM17-2	2.20	37.35	2295	3	52
Q6-4	2.02	37.42	2119	3	51
RH2-1	2.06	37.61	2184	3	55
RH2-2	2.23	37.33	2339	3	56
RH2-3	2.19	37.32	2298	3	56
RH2-4	2.27	37.13	2350	3	49
RQ3-1-1	2.06	37.61	2188	3	55
RQ3-3	2.26	37.13	2365	3	47
RQ3-4	2.06	37.6	2187	3	55

Strains	Genome Size (Mbp)	GC Content (%)	CDS	rRNA	tRNA
RQ3-5	2.01	37.65	2113	3	54
RQ3-6	2.28	37.13	2373	3	52
RQ3-7	2.06	37.61	2189	3	55
Ru15-4	2.30	37.05	2386	2	56
Ru17-11	1.98	37.51	2086	3	56
Ru17-3-1	1.93	37.52	2009	2	57
Ru17-3-2	1.98	37.54	2051	3	53
RU19-3	1.97	37.62	2071	3	52
SL1-1-1	2.02	37.63	2134	3	56
SL1-1-2	2.00	37.63	2116	3	47
SL1-2-1	1.97	37.5	2079	3	52
SL1-4-1	1.99	37.56	2098	4	52
SL1-4-3	1.99	37.56	2093	3	51
SL2-7	1.94	37.62	2042	3	58
SL4-6-1	1.95	37.55	2044	2	52
SMN2-1	1.95	37.59	2046	5	56
SMN2-2	1.98	37.56	2068	3	54
XJ56-4-1	2.19	37.31	2293	4	52
Z2-4	2.03	37.65	2145	3	52

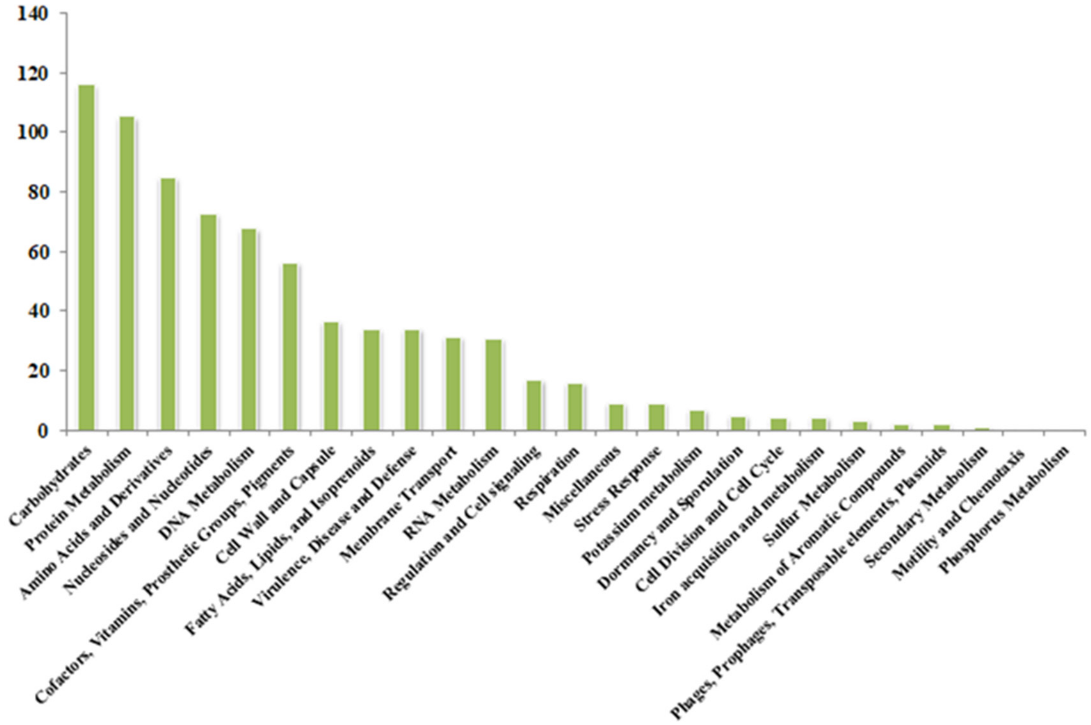

Figure S1. RAST annotation results for 82 strains of *L. kefiranofaciens.*

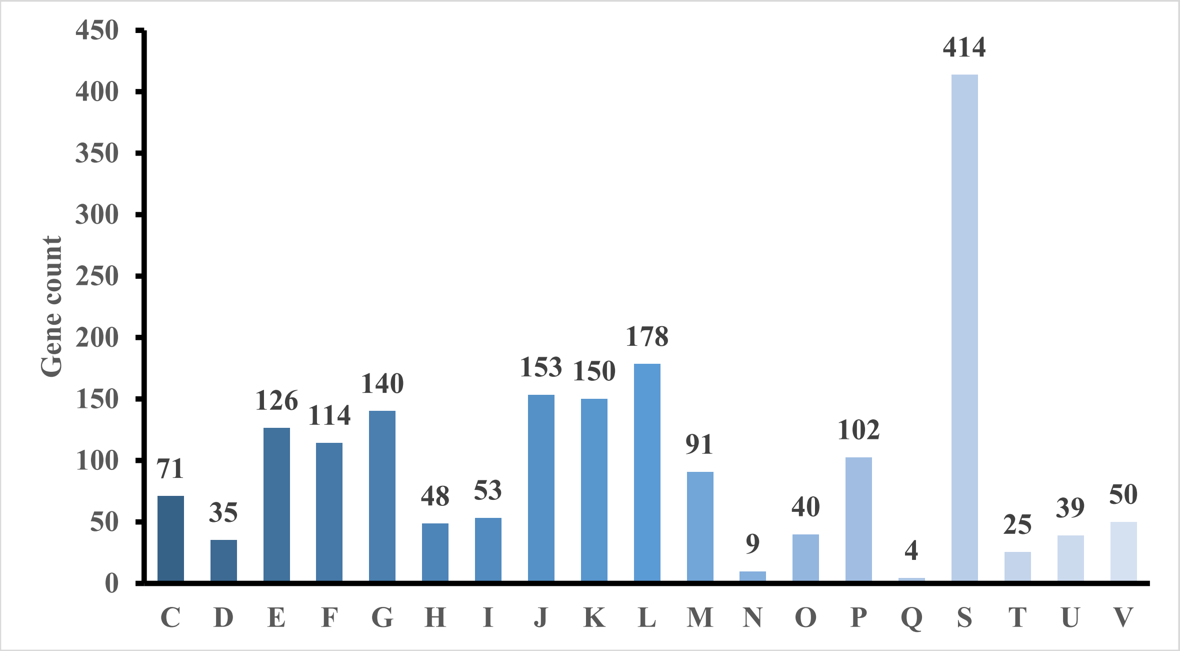

Figure S2. COG annotation results of 82 strains of *L. kefiranofaciens.*

C: Energy production and conversion, D: Cell cycle control, cell division, chromosome Partitioning, E: Amino acid transport and metabolism, F: Nucleotide transport and metabolism, G: Carbohydrate transport and metabolism, H: Coenzyme transport and metabolism, I: Lipid transport and metabolism, J: Translation, ribosomal structure and biogenesis, K: Transcription, L: Replication, recombination and repair, M: Cell wall/membrane/envelope biogenesis, N: Cell motility, O: Posttranslational modification, protein turnover, P: Inorganic ion transport and metabolism, Q: Secondary metabolites biosynthesis, transport and catabolism, T: Signal transduction mechanisms, U: Intracellular trafficking, secretion, and vesicular, V: Defense

mechanisms, S: Function unknown.

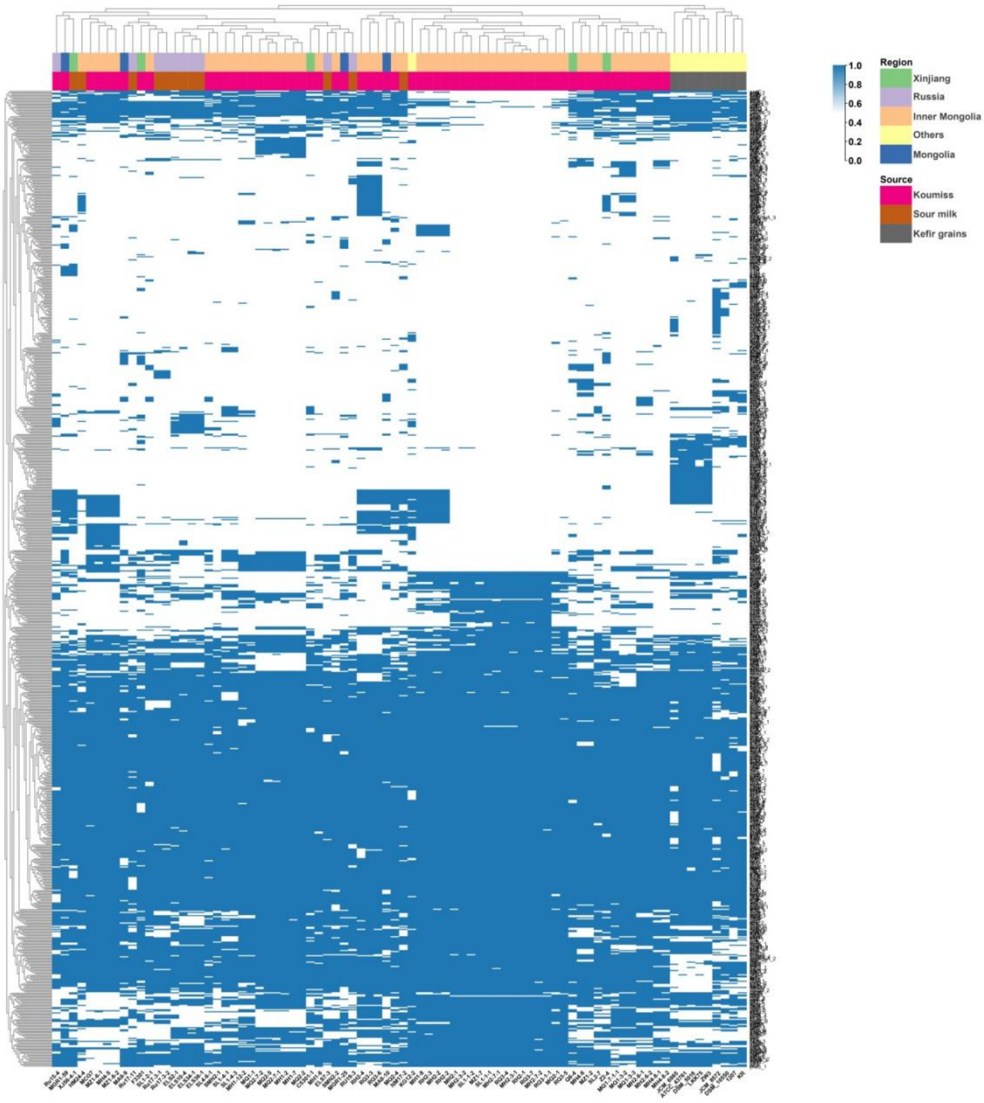

Figure S3. Presence and absence of accessory genes in 82 strains of *L. kefiranofaciens.*
